# Effect of Different Disease-Modifying Therapies on Humoral Response to BNT162b2 Vaccine in Sardinian Multiple Sclerosis Patients

**DOI:** 10.3389/fimmu.2021.781843

**Published:** 2021-12-09

**Authors:** Maristella Pitzalis, Maria Laura Idda, Valeria Lodde, Annalisa Loizedda, Monia Lobina, Magdalena Zoledziewska, Francesca Virdis, Giuseppe Delogu, Federica Pirinu, Maria Giuseppina Marini, Maura Mingoia, Jessica Frau, Lorena Lorefice, Marzia Fronza, Daniele Carmagnini, Elisa Carta, Valeria Orrù, Sergio Uzzau, Paolo Solla, Federica Loi, Marcella Devoto, Maristella Steri, Edoardo Fiorillo, Matteo Floris, Ignazio Roberto Zarbo, Eleonora Cocco, Francesco Cucca

**Affiliations:** ^1^ Institute for Genetic and Biomedical Research, National Research Council, Monserrato, Cagliari, Italy; ^2^ Department of Biomedical Sciences, University of Sassari, Sassari, Italy; ^3^ Regional Multiple Sclerosis Center, Azienda SocioSanitaria Locale (ASSL) Cagliari, Azienda Tutela Salute (ATS) Sardegna, Cagliari, Italy; ^4^ Department of Medical Science and Public Health, University of Cagliari, Cagliari, Italy; ^5^ Department of Biomedical Sciences, University of Sassari - Unit of Clinical Microbiology, Azienza Ospedaliera Universitaria (AOU) Sassari, Sassari, Italy; ^6^ Department of Medical, Surgical and Experimental Sciences, University of Sassari - Neurology Unit, Azienza Ospedaliera Universitaria (AOU) Sassari, Sassari, Italy; ^7^ Osservatorio Epidemiologico Veterinario Regionale, Istituto Zooprofilattico Sperimentale della Sardegna, Cagliari, Italy; ^8^ Dipartimento di Medicina Traslazionale e di Precisione, Università la Sapienza, Rome, Italy

**Keywords:** SARS-CoV-2, multiple sclerosis, humoral immunity, disease-modifying therapy, vaccine efficacy, BNT162b2, COVID-19

## Abstract

**Objectives:**

Vaccination against COVID-19 is highly recommended to patients affected by multiple sclerosis (MS); however, the impact of MS disease-modifying therapies (DMTs) on the immune response following vaccination has been only partially investigated. Here, we aimed to elucidate the effect of DMTs on the humoral immune response to mRNA-based anti-SARS-CoV-2 vaccines in MS patients.

**Methods:**

We obtained sera from 912 Sardinian MS patients and 63 healthy controls 30 days after the second dose of BNT162b2 vaccine and tested them for SARS-CoV-2 response using anti-Spike (S) protein-based serology. Previous SARS-CoV-2 infection was assessed by anti-Nucleocapsid (N) serology. Patients were either untreated or undergoing treatment with a total of 13 different DMTs. Differences between treatment groups comprised of at least 10 patients were assessed by generalized linear mixed-effects model. Demographic and clinical data and smoking status were analyzed as additional factors potentially influencing humoral immunity from COVID-19 vaccine.

**Results:**

MS patients treated with natalizumab, teriflunomide, azathioprine, fingolimod, ocrelizumab, and rituximab showed significantly lower humoral responses compared to untreated patients. We did not observe a statistically significant difference in response between patients treated with the other drugs (dimethyl fumarate, interferon, alemtuzumab and glatiramer acetate) and untreated patients. In addition, older age, male sex and active smoking were significantly associated with lower antibody titers against SARS-CoV-2. MS patients previously infected with SARS-CoV-2 had significantly higher humoral responses to vaccine than uninfected patients.

**Conclusion:**

Humoral response to BNT162b2 is significantly influenced by the specific DMTs followed by patients, as well as by other factors such as previous SARS-CoV-2 infection, age, sex, and smoking status. These results are important to inform targeted strategies to prevent clinically relevant COVID-19 in MS patients.

## Introduction

Coronavirus disease 2019 (COVID-19) is caused by the severe acute respiratory syndrome coronavirus type-2 (SARS-CoV-2) which has spread rapidly worldwide since its first appearance in China in December 2019 (1).

The virus is characterized by high infectivity, particularly for variant B.1.617.2 (delta), which is also sustained by transmission from presymptomatic/asymptomatic carriers. These features of SARS-CoV-2 have led to a large number of infected individuals (more than 250 million molecularly identified individuals) and deaths (more than 5.0 million) through November 2021 [https://www.worldometers.info/coronavirus/].

Elimination of the virus and clinical recovery from COVID-19 mainly relies on the immune response mounted and orchestrated by specific B and T cell subpopulations and mediated by the production of neutralizing antibodies (2, 3). Regarding humoral responses, the most relevant protective antibodies target the SARS-CoV-2 trimeric glycoprotein Spike (S) (4), which mediates the viral entry by interacting with the surface protein angiotensin-converting enzyme 2 expressed in a large set of human cells (5).

Although unprecedented scientific efforts have been made since the pandemic outbreak to limit its clinical impact, and some promising antiviral therapies have begun to be approved [Molnupiravir approved by MHRA], vaccination remains the most effective strategy to contrast the spread of SARS-CoV-2 infection and minimize its clinical impact (6). As of November 2021, there were 24 vaccines approved in different countries and 56 in phase III clinical trials [https://covid19.trackvaccines.org]. The mRNA vaccines BNT162b2 (Pfizer-BioNTech) and mRNA-1273 (Moderna) directed against the spike glycoprotein of SARS-CoV-2 have proven to be safe and especially effective in protecting against infection and severe forms of COVID-19 (7, 8). However, the extent of immune response following vaccination and its efficacy in specific categories of individuals, such as those who are immunocompromised or on immunosuppressive therapies, are only partially clarified.

Disease modifying therapies (DMTs) are immunomodulators and immunosuppressive therapies that can reduce the activity and progression of multiple sclerosis (MS). The extent to which DMTs can influence the response to COVID-19 vaccination in MS patients is a central topic. Recently, a few reports have provided the first preliminary data on humoral responses after Pfizer-BNT162b2-COVID-19 vaccination in MS patients receiving DMTs (9, 10). They suggested that MS patients treated with certain DMTs failed to mount a good protective level of SARS-CoV-2 spike-specific IgG compared to untreated patients. However, the conclusions of these analyses were limited by the small sample sizes and the fact that they did not consider the role of some DMTs and the influence of other elements such as SARS-CoV-2 previous infection or lifestyle on the observed immunologic responses.

Here, we analyzed the humoral response after vaccination with BNT162b2 in a sample of 912 Sardinian MS patients from the Mediterranean island of Sardinia (Italy) which has one of the highest MS prevalence rates in the world (11).

Our findings elucidate the effect of a large spectrum of DMTs and other relevant factors on the humoral responses to BNT162b2, suggesting some categories of MS patients remain at higher risk for SARS-CoV-2 infection following vaccination, and thus informing tailored strategies to prevent COVID-19.

## Methods

### Study Participants

A total of 912 MS patients from the MS clinical centers in Cagliari and Sassari in Sardinia (Italy) were enrolled between April and June 2021. MS patients were diagnosed according to the McDonald criteria. In parallel, 63 healthy individuals from the SardiNIA general population cohort (12) were recruited as a control group.

All patients and controls received two intramuscular injections, 21 days apart, delivered in the deltoid muscle. Each injection contained 30μg of BNT162b2 (0.3ml volume per dose).

Patient information on age, sex, smoking status, disability score, disease subcategory and disease-modifying treatment at the time of vaccination was also collected. The full list of MS patients and healthy controls that received COVID-19 vaccination with clinical and demographic characteristics is available in **Supplemental Table S1**.

### Detection of SARS-CoV-2 IgG Antibodies

Blood samples from the entire study population of 975 individuals were collected approximately 30 days after injection of the second vaccine dose, in vacutainer tubes containing clot activator with gel separator.

Similarly, blood samples were collected for retrospective analyses on the day of the first vaccine injection from a subset of 612 MS patients and from the 63 control subjects.

To avoid time-dependent artifacts, samples were processed within two hours after blood collection, and serum was stored at -80°C until use.

Detection of anti‐SARS‐CoV‐2‐S and anti‐SARS‐CoV‐2‐N antibodies in serum samples was performed using the electrochemiluminescence immunoassays Elecsys^®^ Anti-SARS-CoV-S and Elecsys^®^ Anti-SARS-CoV-N (Roche) on the automated Cobas e-411 analyzer, according to the manufacturer’s instructions. Anti-S results are expressed as units per ml (U/ml).

### Ethics and Data Collection

The studies involving human participants were reviewed and approved by the Ethical Review Boards ATS Sardegna - Prot. N° 2492/CE. Patient data and samples were coded anonymously to ensure confidentiality during sample processing and data analysis. The patients/participants provided their written informed consent to participate in this study.

### Statistical Analysis

Normality of baseline distribution of Anti-S antibodies quantified as described above was preliminary assessed with the Shapiro Wilk test. Categorical variables are presented as number and percentage, quantitative variables are presented as median and interquartile range (IQR).

Considering the nature of the outcome (Anti-S, non-negative count data), differences between groups of patients defined by therapy and negative to Anti-N antibody, were assessed by a negative binomial generalized linear mixed-effects model; the contribution of age, sex, Expanded Disability Status Scale (EDSS), disease duration, previous SARS-CoV-2 infection, and the clinical sampling center were also analyzed. A multivariate model was fitted by including each independent variable with a significant effect in univariate analysis (P < 0.20, nominal p accounting for the number of covariates tested). Only treatments available for at least 10 patients were included in the model. Results are presented as IRR (Incidence Rate Ratio) (13), calculated as the exponential of the regression coefficient. Differences between medians were tested by Mann-Whitney test. All statistical analyses were performed using the R software v.4.1.0. P-values <0.05 were considered as statistically significant.

## Results

### MS Cohort

The MS cohort analyzed in this study included 658 (73.1%) females and 254 (26.9%) males (female-to-male ratio 2.7:1). Regarding the disease forms, 82.7% of patients were relapsing-remitting MS (RRMS), 1.8% primary progressive (PPMS) and 15.5% secondary-progressive (SPMS) (**Table 1** **)**. Of the MS patients, 205 (22.5%) were untreated and 707 (77.5%) treated with various DMTs as follows. The most diffuse common treatments were dimethyl fumarate and interferons (respectively, 22.7% and 19% of the treated patients) while few patients were treated with methotrexate (0.28%) and cladribine (0.84%). In detail, the MS cohort treated with DMTs was composed as follows: 161 patients assumed dimethyl fumarate, 135 interferons, 96 glatiramer acetate, 75 fingolimod, 75 natalizumab, 56 teriflunomide, 42 ocrelizumab, 17 alemtuzumab, 13 rituximab, 6 cladribine and 2 methotrexate. Only one patient was included in a trial with a Bruton’s tyrosine kinase (BTK) inhibitor. Clinical and demographic characteristic of MS patients that received COVID-19 vaccination – stratified for DMTs – are summarized in **Table 2**.

**Table 1:** Clinical and demographic characteristics of MS patients and heathy controls that received COVID-19 vaccination.

	MS patients	Healthy subjects
N.	912	63
Female, n (%)	658 (73.1)	50 (81.0)
Age in yrs, median (IQR)	48.8 (38.8-57.8)	52.1 (42.2-56.1)
Disease duration in yrs, median (IQR)	13.6 (7.6 - 21.6)	_
EDSS, median (IQR)	2.2 (1 - 4.5)	_
RRMS, n (%)	752 (82.6)	_
PPMS, n (%)	16 (1.8)	_
SPMS, n (%)	142 (15.6)	_
Smokers, n (%)^a^	153 (28.6)	_

RRMS, relapsing-remitting multiple sclerosis; EDSS, Expanded Disability Status Scale; IQR, interquartile range.

a Smoking data were available for a subset of 535 MS patients.

**Table 2:** Clinical and demographic characteristic of MS patients that received COVID-19 vaccination stratified by DMTs.

Therapy	N.	Age in yrs. Median (IQR)	% F
ALEM	17	41.3 (36.5-45)	68.8
AZA	28	58.9 (55.2-64.1)	74.1
CLA	6	46.8 (37.8-48)	83.3
DMF	161	44.6 (36.3-51.8)	76.5
FTY	75	45 (37.8-52.1)	74.6
GA	96	53.6 (42.8-59.4)	73.0
IFN	135	47.9 (36.7-54.4)	77.7
NAT	75	36.3 (31.2-46.2)	76.4
OCR	42	43.4 (35.4-52.4)	54.8
RTX	13	53.9 (41.5-56.8)	66.7
TER	56	51.5 (45.6-56.5)	70.9
UNT	205	59 (48.3-65.6)	68.9
MET	2	61.4 (58.8-64.1)	100
BTK	1	57.7	100

DMT, disease modifying therapy; UNT, untreated; ALEM, alemtuzumab; IFN, interferon; GA, glatiramer acetate; DMF, dimethyl fumarate; NAT, natalizumab; CLA, cladribine; TER, teriflunomide; AZA, azathioprine; FTY, fingolimod; RTX, rituximab; OCR, ocrelizumab; MET, methotrexate; BTK, Bruton Tyrosine Kinase trial; IQR, interquartile range; F, female.

### The Impact of Disease-Modifying Therapies on Humoral Responses to BNT162b2 Vaccine

**Figure 1:**
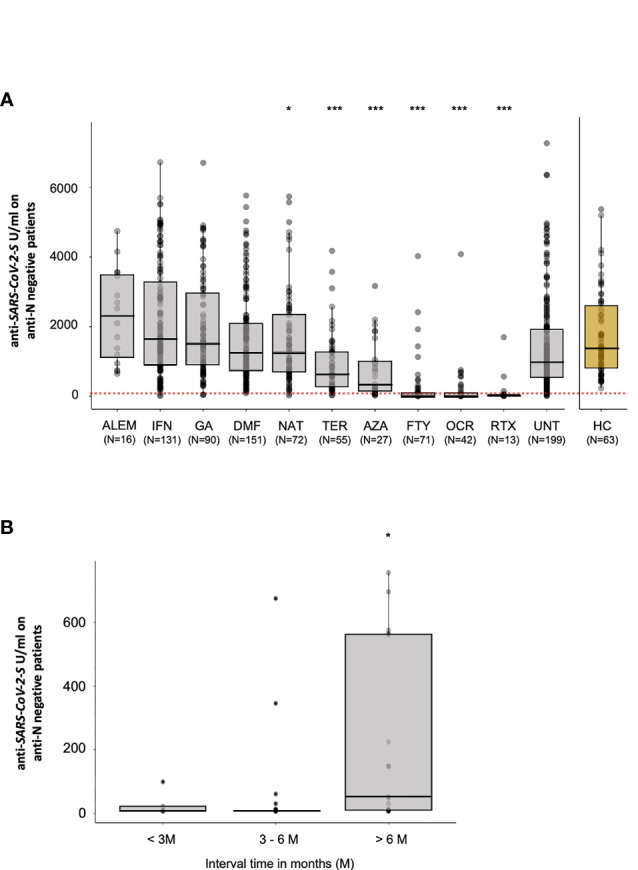
Post-vaccination SARS-CoV-2 antibody response by disease- modifying therapy (DMT). **(A)** Antibody response to SARS-CoV-2 vaccination by DMT in MS patients (UNT, untreated; ALEM, alemtuzumab; IFN, interferon; GA, glatiramer acetate; DMF, dimethyl fumarate; NAT, natalizumab; CLA, cladribine; TER, teriflunomide; AZA, azathioprine; FTY, fingolimod; RTX, rituximab; OCR, ocrelizumab) and healthy control (HC) negative for anti-N antibodies. The red dotted line indicate a cutoff value of 133 U/ml predictive of the presence of neutralizing antibodies according to Resman et al. (14). **(B)** Post-vaccination antibody levels in patients treated with anti-CD20 therapies (rituximab and ocrelizumab) according to the time (< 3-month, 3-6 month and > 6-month) between last anti-CD20 treatment and immunization. Results are reported as boxplots, showing the median value (in bold) and the quartiles as box limits; whiskers at the top and bottom sides represent the overall maximum value and the overall minimum value, respectively. Points outside boxes and whiskers represent outliers. (*P< 0.05; ***P<0.001).

We did not find any significant difference between untreated MS patients and healthy control in anti-S antibodies response (p = 0.51) suggesting not significative effect of MS disease in the response to BNT162b2 vaccine (**Figure 1A** **)**.

We applied a negative binomial generalized linear mixed-effects model in patients negative for Anti-N antibodies production, considering only treatments available for at least 10 patients. We did not find significant difference in Anti-S antibodies production between patients treated with dimethyl fumarate (IRR = 0.867, p = 0.22), interferons (IRR = 1.285, p = 0.06), alemtuzumab (IRR = 1.385, p = 0.22) or glatiramer acetate (IRR = 1.123, p = 0.38) and untreated MS patients (here considered as the reference group); all of them were characterized by high levels of antibodies post-COVID-19 vaccination (**Table 3**).

**Table 3:** Negative binomial generalized linear mixed-effects model of Anti-S-Ab levels in untreated and treated MS patients (Anti-N negative) who received two doses of BNT162b2 vaccine.

Therapy	N.	Median Anti-S in U/ml (IQR 25-75)	IRR	SE	p value
ALEM	16	2310 (1132-3480)	1.385	0.266	0.222
IFN	131	1676 (904-3556)	1.258	0.123	0.063
GA	90	1524 (899-2954)	1.123	0.133	0.383
**UNT**	**199**	**989 (538-1977)**		**reference**	
DMF	151	1250 (761-2108)	0.867	0.118	0.228
NAT	72	1268 (691-2359)	0.725	0.152	0.034
TER	55	619 (283-1273)	0.507	0.157	1.44E-05
AZA	27	342 (165-994)	0.374	0.212	3.26E-06
RTX	13	30.9 (8-52.7)	0.218	0.309	8.36E-07
FTY	71	26.7 (8-87.9)	0.133	0.154	1.89E-39
OCR	42	8 (8-89.7)	0.102	0.177	4.88E-38

SE, Standard Error; IQR, interquantile range; IRR, Incidence Rate Ratio calculated as exp(coefficient) (13); DMT abbreviations as in **Table 2**. In bold we indicated the reference value used for the analysis (UNT).

In contrast, as previously suggested (9, 10), a significantly lower level of Anti-S antibodies production in response to vaccination was observed in MS patients treated with teriflunomide (IRR= 0.51, p = 1.44E-05), azathioprine (IRR = 0.37, p = 3.26E-06), natalizumab (IRR = 0.72, p = 0.034), fingolimod (IRR = 0.13, p = 1.89E-39), ocrelizumab (IRR = 0.10, p = 4.88E-38) and rituximab (IRR = 0.22, p = 8.36E-07), compared to untreated patients (**Table 3**). Of notice is the case of patients treated with natalizumab. Indeed, while the antibodies medians shown in **Figure 1A** seem to indicate a higher response in patients undergoing this treatment compared to untreated patients, results after adjustment for the significant confounding effects of age, sex and EDSS show an opposite (reduced) Anti-S antibodies response. This apparent discrepancy can be explained by the fact that patients treated with natalizumab are overrepresented by female and the youngest among our MS patients, as shown in **Table 2**.

The timing of vaccination relative to the time of last administration of immunosuppressive DMTs may also influence the observed humoral responses. We thus analyzed in more details whether changes in antibody responses are associated with time since last dose of anti-CD20 immunosuppressive drugs, ocrelizumab and rituximab, for which the distribution around median time to discontinuation (132 days for ocrelizumab and 306 days for rituximab) provided sufficient statistical power to detect significant differences. Indeed, in line with other published data (15), we observed that if vaccination occurred 6 months or more after the last dose of anti-CD20 therapy, the antibody responses were significantly upregulated (p = 0.012) compared with shorter time intervals from discontinuation (**Figure 1B**). Recent international recommendations suggest that a 3-month wait represents the optimal interval between the last dose of anti-CD20 treatment and vaccination; our findings can be useful for reevaluating this interval, suggesting that waiting for 6 months could be a better strategy.

The median time to discontinuation of another potentially immunosuppressive DMT, alemtuzumab, before the first dose of vaccine, was 1248 days and in all cases longer than 6 months, thus not providing any useful information about the optimal time to discontinuation before vaccination.

Finally, we evaluated the contribution of DMTs duration on antibody responses by applying a negative binomial generalized linear mixed-effects model. Treatment duration showed a minor but still significant impact on antibody responses (IRR = -0.0008503, p = 0.03).

### The Impact of Prior SARS-CoV-2 Infection on Humoral Responses to BNT162b2 Vaccine

The Nucleocapsid Protein (N) antigens of SARS-CoV-2 are found in the viral core but are not present in the corresponding nucleoside-modified mRNA sequence of BNT162b2 vaccine; thus, immunoglobulins targeting N antigens are detectable in the serum of vaccinated individuals only if they have been recently infected with SARS-CoV-2. To check the effect of previous or concurrent COVID-19 infection on humoral immune response to SARS-CoV-2 vaccination, we quantified anti-SARS-CoV-2 antibodies directed against the N protein in serum samples collected at around 30 days after the injection of the second dose. Only one healthy control was found to be positive for Anti-N antibodies (data not shown). Among the 912 MS patients analyzed we found 38 positives for the N protein, suggesting a previous infection. Of these, 25 had previous record of positive RT−PCR tests on respiratory samples and 22 were found positive to Anti-N antibodies at the time of their first vaccine dose.

**Figure 2:**
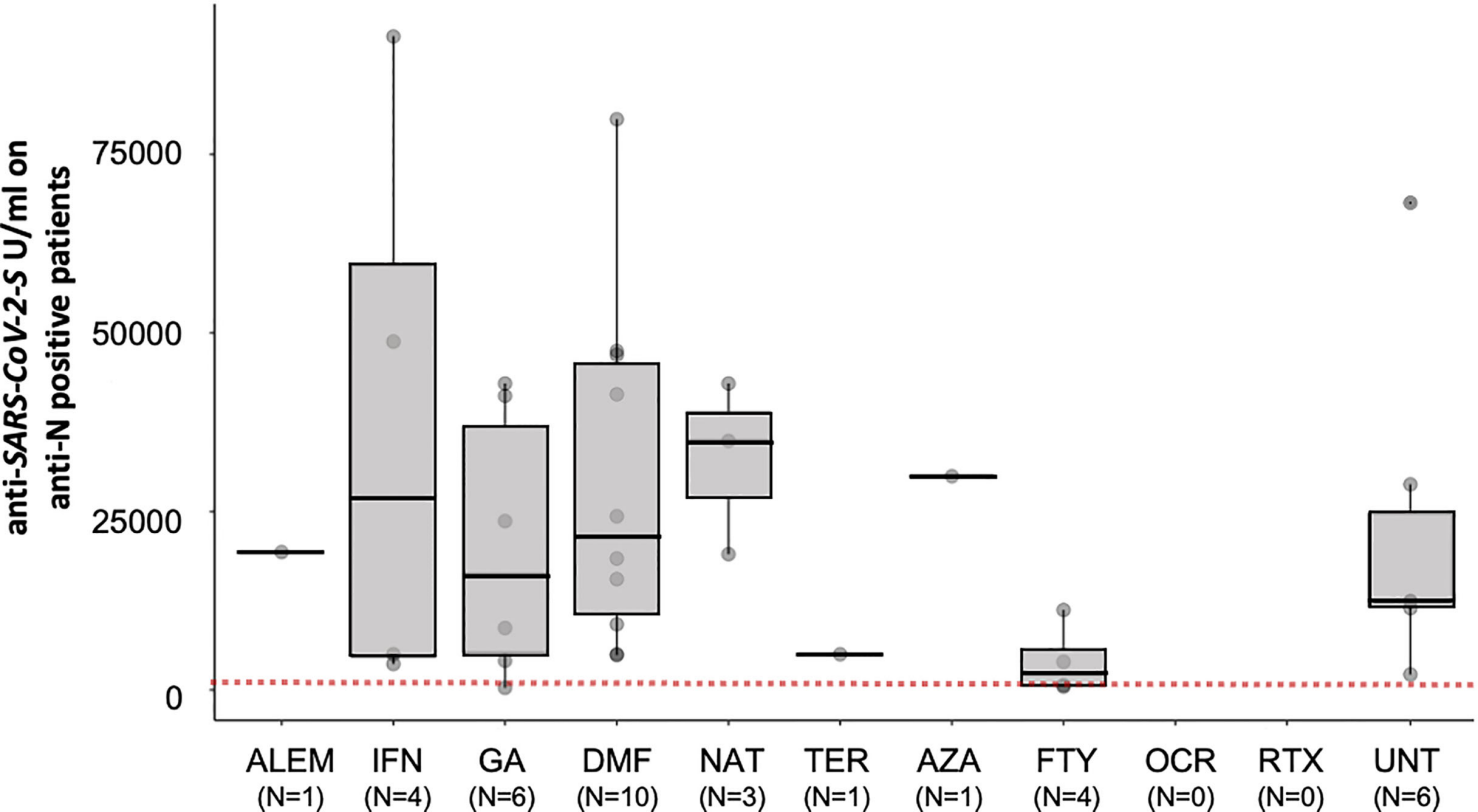
Post-vaccination SARS-CoV-2 antibody response by disease- modifying therapy (DMT) in MS patients positive for anti-N antibodies. Antibody response to SARS-CoV-2 vaccination by DMT in MS patients positive for anti-N antibodies (UNT, untreated; ALEM, alemtuzumab; IFN, interferon; GA, glatiramer acetate; DMF, dimethyl fumarate; NAT, natalizumab; CLA, cladribine; TER, teriflunomide; AZA, azathioprine; FTY, fingolimod; RTX, rituximab; OCR, ocrelizumab). The red dotted lines indicate a cutoff value of 133 U/ml predictive of the presence of neutralizing antibodies according to Resman et al. (14). Results are reported as boxplots, showing the median value (in bold) and the quartiles as box limits; whiskers at the top and bottom sides represent the overall maximum value and the overall minimum value, respectively. Points outside boxes and whiskers represent outliers.

In the 38 MS patients with evidence of a natural exposure to SARS-CoV-2, postvaccination Anti-S antibodies levels were significantly higher than patients which did not experience SARS-CoV-2 infection (medians 14,000 vs. 1,040 U/ml, Mann-Whitney test p = 2.27E-18). The enhanced immune response to vaccine due to natural infection was also observed in patients treated with fingolimod, albeit to a lesser extent than in patients treated with other DMTs (**Figure 2**). Further data are needed to assess the impact of prior or concurrent infection with SARS-CoV-2 on the immune response to the vaccine in patients treated with DMTs in MS patients.

### The Impact of Sex, Age, EDDS, and Smoking on Humoral Responses to BNT162b2 Vaccine

**Figure 3:**
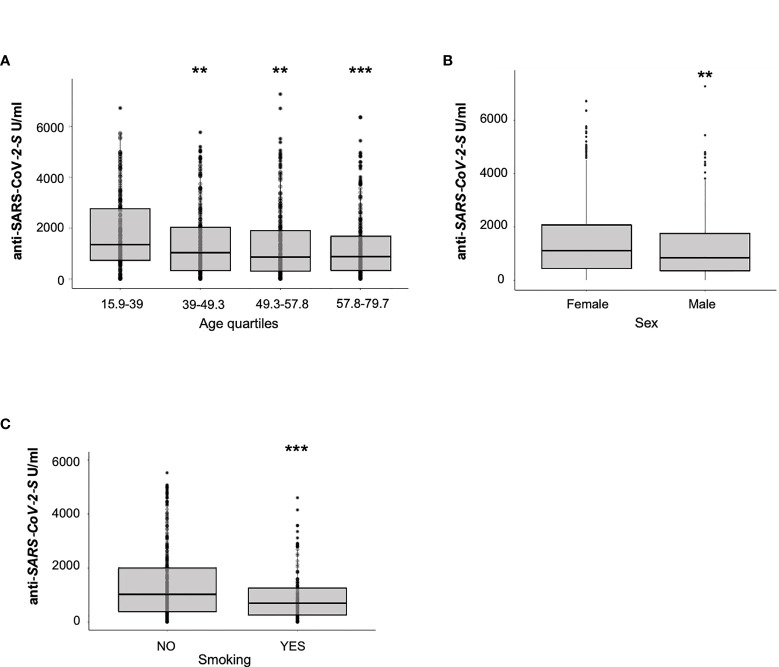
Impact of sex, age and smoking on humoral response to BNT162b2 vaccine. Antibody response to SARS-CoV-2 vaccination by age **(A)**, sex **(B)** and smoking status **(C)** in MS patients is represented. Results are reported as boxplots, showing the median value (in bold) and the quartiles as box limits; the whiskers at the top and bottom sides represent the overall maximum value and the overall minimum value, respectively. Data outside boxes and whiskers represent outliers. (**P<0.01; ***P<0.001).

Analyzing additional factors that may affect interindividual variability in SARS-CoV-2 vaccine responses is extremely important to monitor anti-SARS-CoV-2 vaccine efficacy, particularly in patients affected by MS undergoing DMTs. Our statistical model revealed additional significant effects for age, sex and EDDS, with reduced postvaccination levels of anti-SARS-CoV-2 antibodies directed against the S protein in older (IRR = 0.98, p = 3.85E-03), male individuals (IRR = 0.83, p = 0.021**)** (**Figures 3A, B** **)** or reduced EDDS (IRR=0,93, p = 1.31E-04).

In a recent report, antibodies titers were shown to be lower after COVID-19 vaccination in a healthy cohort of smokers compared to non-smokers (16). We therefore examined the effects of cigarette smoking on humoral response to SARS-CoV-2 vaccine in a subset of 535 MS patients negative for Anti-N antibodies production for whom smoking status was available. Among them, 147 (28.6%) were reported to be active smokers. Our analyses showed that also among MS patients, there was a reduced Anti-S antibodies production in smokers (median = 719 U/ml) compared to nonsmokers (median = 1,054 U/ml) in response to BNT162b2 vaccine (Mann-Whitney test p = 2.0E-04; **Figure 3C**).

## Discussion

In this study we evaluated the early humoral responses to BNT162b2 vaccine in 912 MS patients and 63 healthy individuals from the SardiNIA longitudinal project (12), an autoimmunity prone population (11). The study design allowed us to examine the influence of several DMTs on the levels of total Anti-S antibodies in MS patients. We also considered the influence of other relevant variables, such as a past SARS-CoV-2 infection, as well as age, sex, and smoking.

### Effect of Drugs Used in the Therapy of MS on the Early Humoral Response to SARS-CoV-2 Vaccine

Thirty days after the second dose of vaccine, Anti-S antibodies titers elicited by two doses of BNT162b2 were assessed in MS patients undergoing different DMTs compared to untreated MS patients. Specifically, as already reported in previous smaller studies (9, 10), patients treated with fingolimod, ocrelizumab, and rituximab have much lower humoral responses than untreated patients (**Figure 1A** and **Table 3**). Indeed, anti-CD20 therapies (ocrelizumab and rituximab) and the SP1 modulator fingolimod act directly on B cells, by depleting their availability in circulation or by driving lymphocyte sequestration in the lymph nodes (17, 18). To a lesser but still significant extent, early humoral response was also reduced in patients treated with teriflunomide, natalizumab and azathioprine compared with untreated patients. Teriflunomide is an immunomodulator which causes inhibition of rapidly dividing cells, including activated T and B cells (19). Natalizumab, another immunomodulator, prevents lymphocyte migration to the central nervous system (20). Finally, azathioprine, an immunosuppressive drug used in several autoimmune diseases and in kidney transplants, act through bone-marrow suppression (21).

Patients treated with, interferons, glatiramer acetate, and alemtuzumab, show a higher humoral response than untreated patients, which however does not reach statistical significance in our dataset.

Overall, the effects of many of these DMTs on humoral responses to SARS-CoV-2 are consistent with previous analyses reporting their effects on other vaccines. For instance, preserved immune responses after vaccination against other pathogens (e.g. seasonal influenza, tetanus-diphtheria toxins) have been reported in people treated with beta-interferons and dimethyl fumarate (22); likewise, a reduced responses to vaccinations against other pathogens after treatment with fingolimod and B cell depleting anti-CD20 therapies have been reported (22). Furthermore, our findings on responses after BNT162b2 vaccination in patients treated with alemtuzumab and interferons are in agreement with observations indicating that immune responses to vaccinations are well preserved against a number of vaccines, including the tetanus, Hemophilus influenzae type b and meningococcal group C vaccines (22).

### Previous SARS-CoV2 Infections Enhance the Early Humoral Responses to SARS-Cov-2 Vaccine

One of the most interesting observations of this study is that patients with natural infection, before or soon after the first vaccine injections, had significantly higher humoral responses to vaccine than uninfected patients. Because vaccination aims to stimulate immune responses that mirror natural infection, the finding of enhancement of humoral responses to vaccine in MS patients who have acquired infection, often unnoticed, suggests that for certain DMTs such as fingolimod, an additional boost with a third dose of mRNA vaccine is likely to be effective, as already shown for solid-organ transplant recipients (23) and in the general population (14).

### Older Age, Male Sex, and Smoking Negatively Affect Vaccine Response

In agreement with previous studies (16), we show that in addition to DMTs and prior SARS-CoV-2 infection, both age and sex also influence antibody response to vaccine. Indeed, SARS-CoV-2 antibodies levels against S protein after COVID-19 mRNA vaccine were significantly lower in males and in older patients. These results agree with a very significant reduction in absolute counts of B cells subclasses, including primarily antibody-producing plasma cells, during immunosenescence and with a more pronounced decrease in males than in females (unpublished results from 4,000 individuals of the SardiNIA study ranging from 18 to 105yrs).

Recently, smoking has been shown to be an important risk factor for the development of complications following SARS-CoV-2 infection (24). Hence, we further evaluated the relationship between antibodies titers in response to COVID-19 mRNA vaccine and smoking status in a subgroup of 535 patients; our data indicated a significant reduction in antibodies titers in MS patients who were active smokers. This result is in line with the observation of rapid decrease in antibody titers in smokers after influenza vaccination (25) and more generally with the association of smoking with immune system dysfunction (26).

## Concluding Remarks and Open Questions

The present results highlight the need for an optimal treatment regimen and time schedule for certain DMTs before vaccination to allow repopulation of immune cells and soluble molecules necessary for an appropriate response to the SARS-CoV-2 vaccine, and for resumption of MS therapy after vaccination to prevent a rebound of the autoimmune process underlying the disease. Nevertheless, the suggested administration of a third vaccine dose to safely mimic the enhanced humoral responses observed here in SARS-CoV-2 infected MS patients on immunosuppressive therapies may be the first-line strategy to achieve both effective protection against SARS-CoV2 infection and control of MS by DMT, and will be evaluated in future follow-up analyses.

Still, some open questions remain unsolved. A critical issue is defining a cut-off value for Anti-S antibody levels which correlate with protection against SARS-CoV-2 infection and/or severe COVID-19 disease. In one study, a cut-off value of 133 binding antibody (BA) U/ml for the Elecsy Anti-S assay was found to predict the presence of neutralizing antibodies (NAb) after natural infection (27). Assuming that the same cut-off also applies to sera after vaccination, we can predict the presence of NAb after vaccination in most patients undergoing all DMTs except fingolimod and anti-CD20 therapies. Given the large reduction in total antibodies levels in patients undergoing these latter regimens, it is likely that protection associated with Fc-mediated effector functions of non-NAbs, such as antibody-mediated natural killer cell degranulation and neutrophil phagocytosis, is also severely compromised in the same patients (28). Still, these predictions need to be confirmed by direct neutralization tests.

Another relevant question not addressed in this study is if and which lymphocyte subpopulations are contributing to the immune responses to the vaccine under different DMTs and if a cellular immune reaction triggered by the vaccine provides some protection against infection and disease even in patients with inadequate humoral immune responses. For example, CD4 and CD8 antigen-specific T cell responses were produced after vaccination with mRNA vaccines in a small group of MS patients treated with anti-CD20, with impaired circulating follicular helper T cell responses and enhanced CD8 T cell responses (29). Comprehensive flow cytometry analysis of B and T cells subtypes directed against the Spike protein of SARS-CoV-2 in a larger group of MS patients both untreated and treated with a broader spectrum of DMTs, will be fundamental to fully understand the cellular component of the immune responses after vaccination.

Nevertheless, our current findings showing differential humoral responses to BNT162b2 vaccine in Sardinian MS patients under different DMTs are in agreement with recently published results on MS patients from mainland Italy (15) and have important implications for determining appropriate strategies to ensure an adequate post-vaccination COVID-19 protection while avoiding the risk of a flare-up of inflammatory disease activity.

## Data Availability Statement

The original contributions presented in the study are included in the article/**Supplementary Material**. Further inquiries can be directed to the corresponding authors.

## Ethics Statement

The studies involving human participants were reviewed and approved by the Ethical Review Boards ATS Sardegna - Prot. N° 2492/CE. Patient data and samples were coded anonymously to ensure confidentiality during sample processing and data analysis. The patients/participants provided their written informed consent to participate in this study.

## Author Contributions

MP, MLI, IZ, and EC organized and participated to sample collection. VL, AL, MZ, FV, GD, FP, MGM, MM, JF, LR, MFr, DC and ECa contribute to sample collection. ML provided technical support. SU, VO, EF, PS and FL provided expertise and critical feedback. MD, MS, and MFl performed bioinformatic analysis. MP, MI, IZ, EC, and FC conceived the study. MP and MI wrote the first draft of the manuscript. MP, MI, MFl, MD MS, IZ, EC, and FC revised the manuscript. All authors contributed to manuscript revision, read, and approved the submitted version.

## Funding

The study was supported by the Italian Foundation for Multiple Sclerosis- FISM (Grant N. 2021/S/2).

## Conflict of Interest

The authors declare that the research was conducted in the absence of any commercial or financial relationships that could be construed as a potential conflict of interest.

## Publisher’s Note

All claims expressed in this article are solely those of the authors and do not necessarily represent those of their affiliated organizations, or those of the publisher, the editors and the reviewers. Any product that may be evaluated in this article, or claim that may be made by its manufacturer, is not guaranteed or endorsed by the publisher.

## Abbreviations

MS, Multiple sclerosis; DMTs, disease-modifying therapies; SARS-CoV-2, severe acute respiratory syndrome coronavirus type-2; COVID-19, Coronavirus disease 2019; Ab, Antibody; S, Spike; N, Nucleocapsid.

## References

[1] Seyed Hosseini E Riahi Kashani N Nikzad H Azadbakht J Hassani Bafrani H Haddad Kashani H. The Novel Coronavirus Disease-2019 (COVID-19): Mechanism of Action, Detection and Recent Therapeutic Strategies. Virology (2020) 551:1–9. doi: 10.1016/j.virol.2020.08.011 33010669 PMC7513802

[2] Toor SM Saleh R Sasidharan Nair V Taha RZ Elkord E. T-Cell Responses and Therapies Against SARS-CoV-2 Infection. Immunology (2021) 162:30–43. doi: 10.1111/imm.13262 32935333 PMC7730020

[3] Schultheiß C Paschold L Simnica D Mohme M Willscher E von Wenserski L. Next-Generation Sequencing of T and B Cell Receptor Repertoires From COVID-19 Patients Showed Signatures Associated With Severity of Disease. Immunity (2020) 53:442–455.e4. doi: 10.1016/j.immuni.2020.06.024 32668194 PMC7324317

[4] Cao W Li T. COVID-19: Towards Understanding of Pathogenesis. Cell Res (2020) 30:367–9. doi: 10.1038/s41422-020-0327-4 PMC7186532 32346073

[5] Lan J Ge J Yu J Shan S Zhou H Fan S. Structure of the SARS-CoV-2 Spike Receptor-Binding Domain Bound to the ACE2 Receptor. Nature (2020) 581:215–20. doi: 10.1038/s41586-020-2180-5 32225176

[6] Hodgson SH Mansatta K Mallett G Harris V Emary KRW Pollard AJ. What Defines an Efficacious COVID-19 Vaccine? A Review of the Challenges Assessing the Clinical Efficacy of Vaccines Against SARS-CoV-2. Lancet Infect Dis (2021) 21:e26–35. doi: 10.1016/S1473-3099(20)30773-8 PMC7837315 33125914

[7] Thomas SJ Moreira ED Kitchin N Absalon J Gurtman A Lockhart S. Safety and Efficacy of the BNT162b2 mRNA Covid-19 Vaccine Through 6 Months. N Engl J Med (2021) 385:1761–73. doi: 10.1056/NEJMoa2110345 PMC8461570 34525277

[8] Baden LR El Sahly HM Essink B Kotloff K Frey S Novak R. Efficacy and Safety of the mRNA-1273 SARS-CoV-2 Vaccine. N Engl J Med (2021) 384:403–16. doi: 10.1056/NEJMoa2035389 PMC7787219 33378609

[9] Achiron A Dolev M Menascu S Zohar D-N Dreyer-Alster S Miron S. COVID-19 Vaccination in Patients With Multiple Sclerosis: What We Have Learnt by February 2021. Mult Scler (2021) 27:864–70. doi: 10.1177/13524585211003476 PMC8114441 33856242

[10] Bigaut K Kremer L Fabacher T Lanotte L Fleury M-C Collongues N. Impact of Disease-Modifying Treatments of Multiple Sclerosis on Anti–SARS-CoV-2 Antibodies: An Observational Study. Neurol Neuroimmunol Neuroinflamm (2021) 8:e1055. doi: 10.1212/NXI.0000000000001055 34321333 PMC8362343

[11] Urru SA Antonelli A Sechi GM. MS Working Group. Prevalence of Multiple Sclerosis in Sardinia: A Systematic Cross-Sectional Multi-Source Survey. Mult Scler (2020) 26:372–80. doi: 10.1177/1352458519828600 30793660

[12] Pilia G Chen W-M Scuteri A Orrú M Albai G Dei M. Heritability of Cardiovascular and Personality Traits in 6,148 Sardinians. PloS Genet (2006) 2:e132. doi: 10.1371/journal.pgen.0020132 16934002 PMC1557782

[13] Hilbe J. Negative Binomial Regression. Cambridge; New York: Cambridge University Press (2011).

[14] Falsey AR Frenck RW Walsh EE Kitchin N Absalon J Gurtman A. SARS-CoV-2 Neutralization With BNT162b2 Vaccine Dose 3. N Engl J Med (2021) 385:1627–9. doi: 10.1056/NEJMc2113468 34525276 PMC8461567

[15] Sormani MP Inglese M Schiavetti I Carmisciano L Laroni A Lapucci C. Effect of SARS-CoV-2 mRNA Vaccination in MS Patients Treated With Disease Modifying Therapies. EBioMedicine (2021) 72:103581. doi: 10.1016/j.ebiom.2021.103581 34563483 PMC8456129

[16] Watanabe M Balena A Tuccinardi D Tozzi R Risi R Masi D. Central Obesity, Smoking Habit, and Hypertension Are Associated With Lower Antibody Titres in Response to COVID-19 mRNA Vaccine. Diabetes Metab Res Rev (2021) 6:e3465. doi: 10.1002/dmrr.3465 PMC8209952 33955644

[17] Hauser SL Bar-Or A Comi G Giovannoni G Hartung H-P Hemmer B. Ocrelizumab Versus Interferon Beta-1a in Relapsing Multiple Sclerosis. N Engl J Med (2017) 376:221–34. doi: 10.1056/NEJMoa1601277 28002679

[18] Matloubian M Lo CG Cinamon G Lesneski MJ Xu Y Brinkmann V. Lymphocyte Egress From Thymus and Peripheral Lymphoid Organs Is Dependent on S1P Receptor 1. Nature (2004) 427:355–60. doi: 10.1038/nature02284 14737169

[19] Klotz L Eschborn M Lindner M Liebmann M Herold M Janoschka C. Teriflunomide Treatment for Multiple Sclerosis Modulates T Cell Mitochondrial Respiration With Affinity-Dependent Effects. Sci Transl Med (2019) 11:eaao5563. doi: 10.1126/scitranslmed.aao5563 31043571

[20] Miller DH Khan OA Sheremata WA Blumhardt LD Rice GPA Libonati MA. A Controlled Trial of Natalizumab for Relapsing Multiple Sclerosis. N Engl J Med (2003) 348:15–23. doi: 10.1056/NEJMoa020696 12510038

[21] Hauser SL Cree BAC. Treatment of Multiple Sclerosis: A Review. Am J Med (2020) 133:1380–90.e2. doi: 10.1016/j.amjmed.2020.05.049 32682869 PMC7704606

[22] Ciotti JR Valtcheva MV Cross AH. Effects of MS Disease-Modifying Therapies on Responses to Vaccinations: A Review. Multiple Sclerosis Relat Disord (2020) 45:102439. doi: 10.1016/j.msard.2020.102439 PMC7395588 32769063

[23] Kamar N Abravanel F Marion O Couat C Izopet J Del Bello A. Three Doses of an mRNA Covid-19 Vaccine in Solid-Organ Transplant Recipients. N Engl J Med (2021) 385:661–2. doi: 10.1056/NEJMc2108861 PMC8262620 34161700

[24] COVID-19 Host Genetics Initiative. Mapping the Human Genetic Architecture of COVID-19. Nature (2021). doi: 10.1038/s41586-021-03767-x PMC8674144 34237774

[25] Skowronski DM Tweed SA De Serres G. Rapid Decline of Influenza Vaccine–Induced Antibody in the Elderly: Is It Real, or Is It Relevant? J Infect Dis (2008) 197:490–502. doi: 10.1086/524146 18275271

[26] 23andMe Research Team HUNT All-In Psychiatry Liu M Jiang Y Wedow R Li Y. Association Studies of Up to 1.2 Million Individuals Yield New Insights Into the Genetic Etiology of Tobacco and Alcohol Use. Nat Genet (2019) 51:237–44. doi: 10.1038/s41588-018-0307-5 PMC6358542 30643251

[27] Resman Rus K Korva M Knap N Avšič Županc T Poljak M. Performance of the Rapid High-Throughput Automated Electrochemiluminescence Immunoassay Targeting Total Antibodies to the SARS-CoV-2 Spike Protein Receptor Binding Domain in Comparison to the Neutralization Assay. J Clin Virol (2021) 139:104820. doi: 10.1016/j.jcv.2021.104820 33865031 PMC8035809

[28] Atyeo C Fischinger S Zohar T Slein MD Burke J Loos C. Distinct Early Serological Signatures Track With SARS-CoV-2 Survival. Immunity (2020) 53:524–32.e4. doi: 10.1016/j.immuni.2020.07.020 32783920 PMC7392190

[29] Apostolidis SA Kakara M Painter MM Goel RR Mathew D Lenzi K. Cellular and Humoral Immune Responses Following SARS-CoV-2 mRNA Vaccination in Patients With Multiple Sclerosis on Anti-CD20 Therapy. Nat Med (2021). doi: 10.1038/s41591-021-01507-2 PMC8604727 34522051

